# Supplementary material for: Analysis of biliary MICRObiota in hepatoBILIOpancreatic diseases compared to healthy people [MICROBILIO]: Study protocol
Source: PLoS One. 2020 Nov 19;15(11):e0242553. doi: 10.1371/journal.pone.0242553 (PMC7676666; doi:10.1371/journal.pone.0242553)
Supplement: S1 File — (DOCX) [file pone.0242553.s001.docx]

INFORMED CONSENT FORM

___________________________________________________________________

We invite you to participate in this project and collaborate in the development of this research. Below is more information:

**I - DATA ON SCIENTIFIC RESEARCH**

**1.** TITLE OF THE RESEARCH PROTOCOL: “ANALYSIS OF THE BILIARY MICROBIOTA IN HEPATOBILIOPANCREATIC DISEASES COMPARED TO HEALTHY PEOPLE”

**2.** RESEARCHER: Alberto Meyer

POSITION / FUNCTION: Assistant Physician

DEPARTMENT / INSTITUTE: Liver and Digestive System Transplant Service, Hospital das Clínicas, FMUSP.

**II - REGISTRATION OF THE RESEARCHER'S EXPLANATIONS TO THE PATIENT OR ITS LEGAL REPRESENTATIVE ABOUT THIS RESEARCH**

**1.** JUSTIFICATION AND OBJECTIVES OF THE STUDY.

Our body is inhabited by a large number of bacteria, which together we call “microbiome”. The microbiome is very important for our health, and perhaps its composition is important in the development of diseases in the liver, gallbladder and pancreas. Therefore, in this project, we will try to identify which bacteria are found in the biliary microbiome of sick and healthy people to compare them and find out if they have any role in the development of diseases. We are requesting your authorization so that we can collect your bile to continue with the studies.

**2.** DESCRIPTION OF THE PROCEDURES AND METHODS THAT WILL BE USED Patients who will participate in this research will be liver donors or those who will undergo an endoscopic retrograde cholangiopancreatography (ERCP) examination, as requested by your doctor. We will take advantage of these situations to request the collection of samples of 1ml (20 drops) of bile to research the microbiome. We will also make use of some data from your medical record without any identification of your name.

**3.** DESCRIPTION OF DISCOMFORT AND RISKS ARISING FROM PARTICIPATION IN RESEARCH

There will be no discomfort and risks expected during the collection of bile in any of the procedures.

**4.** EXPECTED BENEFITS FOR THE PARTICIPANT.

There is no immediate benefit for the participant, but the results of this study may be of benefit to future patients with diseases of the liver, gallbladder and pancreas.

**5.** CLARIFICATION ON THE MONITORING AND ASSISTANCE TO WHICH RESEARCH PARTICIPANTS WILL BE ENTITLED

All patients will receive monitoring and assistance during the course of this research

**6.** WITHDRAWAL OF CONSENT OR REFUSAL TO PARTICIPATE

Freedom is guaranteed to refuse to participate or withdraw consent and to stop participating in this study at any time, without prejudice to the continuity of its treatment at the institution, without any penalty, of secrecy and privacy.

**7.** SECOND WAY OF THE CONSENT

The participant is guaranteed to receive one copy of this free and informed consent term, in which the researcher and the participant must initial all copies.

**8.** REFUND AND INDEMNITY

On the same day of the consultation with the Liver Transplant and / or Endoscopy team, or during hospitalization before the transplant or ERCP, the consent term can be signed. There will be no financial compensation related to your participation.

In case of personal injury, directly caused by the procedures or treatments proposed in this study (proven causal link), the participant is entitled to medical treatment at the institution, as well as the legally established indemnities. Expenses are not expected, as patients will participate in the research during a previously scheduled routine consultation

**9.** STORAGE OF BIOLOGICAL MATERIAL

Once this study is completed, your biological material will receive a code and will be filed in our laboratory anonymously, without your name or any data that can identify you, and can be used for other academic studies, without commercial purpose, as long as approved by the Research Ethics Committee, in accordance with the guidelines of the national body that coordinates the principles of research in our country, “Comissão Nacional de Ética em Pesquisa” (CONEP).

**10.** GUARANTEE OF ACCESS TO THE RESEARCHER.

At any stage of the study, you will have access to the professionals responsible for the research to clarify any doubts. The main researcher is Dr. Alberto Meyer who can be found at: Av. Dr. Enéas de Carvalho Aguiar, 255, 9o andar, sala 9113/9114, tel: +55+11+2661-3323 or +55+11+2661-3324. If you have any doubts or doubts about research ethics please contact the Research Ethics Committee - Rua Ovídio Pires de Campos, 225 - 5th floor - tel: +55+11+2661-7585, E-mail: cappesq.adm@hc.fm.usp.br.

I believe I have been sufficiently informed about the information I read or that was read to me describing the study ANALYSIS OF THE BILIARY MICROBIOTA IN HEPATOBILIOPANCREATIC DISEASES COMPARED TO HEALTHY PEOPLE.

I discussed with Dr. Alberto Meyer or a member of his team about my decision to participate in this study. It was clear to me what the purposes of the study are, the procedures to be performed, their discomforts and risks, the guarantees of confidentiality and permanent clarifications. It was also clear that my participation is free of charge and that I have guaranteed access to hospital treatment when necessary. I voluntarily agree to participate in this study and I will be able to withdraw my consent at any time, before or during it, without penalty or loss of any benefit that I may have acquired, or in my service at HCFMUSP. I sign this consent form and receive a copy initialed by the researcher.

________________________________________

Patient Signature / Legal Representative

Date ____ /____ /____

_____________________________________________________

Patient Name / Legal Representative

_____________________________________________________

Signature of the person responsible for the study

Date ____/____ /____
