# Supplementary material for: Analysis of biliary MICRObiota in hepatoBILIOpancreatic diseases compared to healthy people [MICROBILIO]: Study protocol
Source: PLoS One. 2020 Nov 19;15(11):e0242553. doi: 10.1371/journal.pone.0242553 (PMC7676666; doi:10.1371/journal.pone.0242553)
Supplement: S2 File — (DOCX) [file pone.0242553.s002.docx]

TERMO DE CONSENTIMENTO LIVRE E ESCLARECIDO

__________________________________________________________________________

Convidamos o(a) Sr.(a) para participar deste projeto e colaborar para o desenvolvimento desta pesquisa. Seguem abaixo mais informações:

**I - DADOS SOBRE A PESQUISA CIENTÍFICA**

**1**. TÍTULO DO PROTOCOLO DE PESQUISA: “ANÁLISE DA MICROBIOTA BILIAR EM DOENÇAS HEPATOBILIOPANCREÁTICAS EM COMPARAÇÃO COM PESSOAS SADIAS”

**2**. PESQUISADOR: Alberto Meyer

CARGO/FUNÇÃO: Médico Assistente

DEPARTAMENTO/INSTITUTO: Serviço de Transplante de Fígado e Órgãos do Aparelho Digestivo do Hospital das Clínicas da FMUSP.

**II - REGISTRO DAS EXPLICAÇÕES DO PESQUISADOR AO PACIENTE OU SEU REPRESENTANTE LEGAL SOBRE ESTA PESQUISA**

**1**. JUSTIFICATIVA E OBJETIVOS DO ESTUDO.

O nosso corpo é habitado por um grande número de bactérias, o que conjuntamente chamamos de “microbioma”. O microbioma é muito importante para nossa saúde, e talvez sua composição tenha importância no desenvolvimento de doenças no fígado, vesícula biliar e pâncreas. Por isso, neste projeto, vamos tentar identificar quais bactérias se encontram no microbioma biliar de pessoas doentes e pessoas sadias para compará-las e descobrir se elas têm algum papel no desenvolvimento das doenças. Nós estamos solicitando sua autorização para podermos coletar sua bile para seguirmos com os estudos.

**2**. DESCRIÇÃO DOS PROCEDIMENTOS E MÉTODOS QUE SERÃO EMPREGADOS Os pacientes que participarão desta pesquisa serão doadores de fígado ou aqueles que irão se submeter a um exame de colangiopancreatografia retrógrada endoscópica (CPRE), já solicitado por seu médico. Iremos aproveitar essas situações para solicitar a coleta de amostras de 1ml (20 gotas) de bile para pesquisar o microbioma. Também faremos uso de alguns dados de seu prontuário sem qualquer identificação de seu nome.

**3.** DESCRIÇÃO DOS DESCONFORTOS E RISCOS DECORRENTES DA PARTICIPAÇÃO NA PESQUISA

Não ocorrerá desconfortos e riscos esperados durante a coleta da bile em nenhum dos procedimentos.

**4**. BENEFÍCIOS ESPERADOS PARA O PARTICIPANTE.

Não existe um benefício imediato para o participante, contudo os resultados desse estudo poderão trazer benefícios para futuros pacientes com doenças no fígado, na vesícula biliar e no pâncreas.

**5**. ESCLARECIMENTO SOBRE A FORMA DE ACOMPANHAMENTO E ASSISTÊNCIA A QUE TERÃO DIREITO OS PARTICIPANTES DA PESQUISA

todos os pacientes receberão acompanhamento e assistência durante o desenrolar desta pesquisa

**6.** RETIRADA DO CONSENTIMENTO OU RECUSA DE PARTICIPAÇÃO

É garantida a liberdade para recusar-se a participar ou retirar o consentimento e deixar de participar deste estudo a qualquer momento, sem qualquer prejuízo à continuidade de seu tratamento na instituição, sem penalização alguma, de sigilo e privacidade.

**7.** SEGUNDA VIA DO TERMO DE CONSENTIMENTO

É garantido ao participante o recebimento de uma via deste termo de consentimento livre e esclarecido, em que o pesquisador e o participante devem rubricar todas as vias.

**8.** RESSARCIMENTO E INDENIZAÇÃO

No mesmo dia da consulta com a equipe de Transplante de Fígado e/ou Endoscopia, ou durante internação antes do transplante ou CPRE, poderá ser assinado o TCLE. Não haverá compensação financeira relacionada à sua participação.

Em caso de dano pessoal, diretamente causado pelos procedimentos ou tratamentos propostos neste estudo (nexo causal comprovado), o participante tem direito a tratamento médico na instituição, bem como às indenizações legalmente estabelecidas. Não são esperadas despesas, uma vez que os pacientes participarão da pesquisa durante consulta de rotina previamente agendada

**9.** ARMAZENAMENTO DE MATERIAL BIOLÓGICO

Uma vez finalizado este estudo o seu material biológico receberá um código e será arquivado em nosso laboratório de forma anônima, sem seu nome ou qualquer dado que possa identificá-lo, e poderá ser utilizado para outros estudos acadêmicos, sem finalidade comercial, desde que aprovado pelo Comitê de Ética em Pesquisa (CEP), em conformidade com as orientações do órgão nacional que coordena os princípios de pesquisa em nosso país, a CONEP.

**10.** GARANTIA DE ACESSO AO PESQUISADOR.

Em qualquer etapa do estudo, você terá acesso aos profissionais responsáveis pela pesquisa para esclarecimento de eventuais dúvidas. O investigador principal é o Dr. Alberto Meyer que pode ser encontrado no endereço: Av. Dr. Enéas de Carvalho Aguiar, 255, 9^o^ andar, sala 9113/9114, tel: 2661-3323 ou 2661-3324. Se você tiver alguma consideração ou dúvida sobre a ética da pesquisa entre em contato com o Comitê de Ética em Pesquisa (CEP) – Rua Ovídio Pires de Campos, 225 – 5º andar – tel: 2661-7585, Email: [cappesq.adm@hc.fm.usp.br](mailto:cappesq.adm@hc.fm.usp.br).

Acredito ter sido suficientemente informado a respeito das informações que li ou que foram lidas para mim descrevendo o estudo ANÁLISE DA MICROBIOTA BILIAR EM DOENÇAS HEPATOBILIOPANCREÁTICAS EM COMPARAÇÃO COM PESSOAS SADIAS.

Eu discuti com o Dr. Alberto Meyer ou algum membro de sua equipe sobre a minha decisão em participar desse estudo. Ficaram claros para mim quais são os propósitos do estudo, os procedimentos a serem realizados, seus desconfortos e riscos, as garantias de confidencialidade e de esclarecimentos permanentes. Ficou claro também que minha participação é isenta de despesas e que tenho garantia de acesso a tratamento hospitalar quando necessário. Concordo voluntariamente em participar deste estudo e poderei retirar o meu consentimento a qualquer momento, antes ou durante o mesmo, sem penalidades, prejuízo ou perda de qualquer benefício que eu possa ter adquirido, ou no meu atendimento no HCFMUSP. Assino este termo de consentimento e recebo um via rubricada pelo pesquisador.

____________________________________________________

Assinatura do Paciente/ Representante Legal

Data         /       /

_____________________________________________________

Nome do Paciente/ Representante Legal

_____________________________________________________

Assinatura do responsável pelo estudo

Data        /       /
