# Supplementary material for: Analysis of biliary MICRObiota in hepatoBILIOpancreatic diseases compared to healthy people [MICROBILIO]: Study protocol
Source: PLoS One. 2020 Nov 19;15(11):e0242553. doi: 10.1371/journal.pone.0242553 (PMC7676666; doi:10.1371/journal.pone.0242553)
Supplement: Attachment — Submitted filename: Response to the Reviewers.docx [file pone.0242553.s003.docx]

Dear reviewers,

We are pleased and thankful with your response on our submittion of the study protocol “**Analysis of biliary microbiota in hepatobiliopancreatic diseases compared to healthy people [MICROBILIO]: study protocol**”. Here we present our rebuttal letter, answering each point raised by the academic editor and reviewers. The journal comments are in **bold;** our responses are in normal type.

**1. Please ensure that your manuscript meets PLOS ONE's style requirements, including those for file naming. The PLOS ONE style templates can be found at**

[**https://journals.plos.org/plosone/s/file?id=wjVg/PLOSOne_formatting_sample_main_body.pdf**](https://journals.plos.org/plosone/s/file?id=wjVg/PLOSOne_formatting_sample_main_body.pdf) **and** [**https://journals.plos.org/plosone/s/file?id=ba62/PLOSOne_formatting_sample_title_authors_affiliations.pdf**](https://journals.plos.org/plosone/s/file?id=ba62/PLOSOne_formatting_sample_title_authors_affiliations.pdf)

A: Dear reviewers, the necessary style changes were made, according to the requirements of the journal, following the models presented.

**2. Your ethics statement should only appear in the Methods section of your manuscript. If your ethics statement is written in any section besides the Methods, please delete it from any other section.**

A: Dear reviewers, the ethics statement was previously on a separate topic, but it has been modified so that it appears in the Methods section, as requested.

**3.We note that your study will involve tissue/organ transplantation. Please provide the following information regarding tissue/organ donors for transplantation cases analyzed in your study. Even if your study will be retrospective or focused on transplant recipient outcomes, the requested details about the donors are needed to clarify whether the procedures will meet international standards for tissue/organ transplantation.**

**1. Please state in your response letter and ethics statement whether the transplant cases for this study will involve any vulnerable populations; for example, tissue/organs from prisoners, subjects with reduced mental capacity due to illness or age, or minors.**

**- If a vulnerable population will be used, please describe the population, justify the decision to use tissue/organ donations from this group, and clearly describe what measures were taken in the informed consent procedure to assure protection of the vulnerable group and avoid coercion.**

**- If a vulnerable population will not be used, please state in your ethics statement, “None of the transplant donors will be from a vulnerable population and all donors or next of kin will have provided written informed consent that was freely given.”**

A: Dear reviewers, none of the transplant donors will be from a vulnerable population and all donors or next of kin will have provided written informed consent that was freely given, being duly established in the Ethics statement, as requested.

**2. In the ethods, please provide detailed information about the procedure by which informed consent will be obtained from organ/tissue donors or their next of kin. In addition, please provide a blank example of the form used to obtain consent from donors, and an English translation if the original is in a different language.**

A: Dear reviewers, in Methods, under the topic Ethics approval and consent to participate, it was informed how consent will be obtained from the organ donors. In this submission, the model of the informed consent form was also attached in its original language (Portuguese) and its English translation in Supporting Infomation.

**3. Please discuss whether medical costs will be covered or other cash payments will be provided to the family of the donor. If so, please specify the value of this support (in local currency and equivalent to U.S. dollars).**

A: As for costs and payments to donors and families, no payment will be offered and participation will be voluntary. In addition, there will be no medical costs, as all procedures will be performed in the public health system, in the teaching hospital - Hospital das Clínicas University of Sao Paulo School of Medicine (HCFMUSP), entirely free of charge.

**6. Review Comments to the Author**

**1. Sample size/Power: The sample size/power statement should explicitly mention the "name" of the statistical test used, and the "response" that was used to calculate this. The authors' mentioned effect sizes, but never mentioned the name of the test, whether it was one-tailed, or two-tailed, etc. a two-tailed test. Better to be specific (what test?).**

A: Thank you, dear reviewers, for your comment, highlighting this mistake. It has already been corrected and added in Methods, in the sub-item Statistical Analysis, the name of the test, and its due characteristics, as requested, better specifying the details, as the reviewer can check.

**2. Writing style: Even before presenting the sample size/power, I found lot of words and sentences used in describing 16S rRNA method, but I never found "what is the response" they will be using? Microbiome data is usually compositional in nature; see article below:**

[**https://www.frontiersin.org/articles/10.3389/fmicb.2017.02224/full**](https://www.frontiersin.org/articles/10.3389/fmicb.2017.02224/full)

**The writeup needs to clearly describe the type of responses to be used, specifically, are they using the compositional data, or some "summary" of the compositional outcomes to conduct the comparison between disease and controls.**

**There is a pretty robust literature on conducting microbiome data analysis using compositional data, using standard freeware (like R); the authors are suggested to consider that, versus doing some rudimentary/half-hearted analysis. I strongly suggest addition of a qualified statistician/bioinformatician within the research team to produce nice data analysis results, and not some point and click.**

A: Thank you for your detailed comments and for providing the reference. As explained in the Methods, our raw data will be sequencing reads of two kinds: rRNA 16S and total (shotgun) DNA. As such, as the reference cited says, these data are compositional. All of the methods and statistical tests that we plan to use take this into account. One of the authors of the present study is Dr. J.C. Setubal, who is the team’s bioinformatician.

**3. Abstract/Statistical Analysis Methods: That brings me to the abstract & statistical analysis proposed. I see names of tests, like Student's t, and Man-Whitney tests. Again, it reads weird, when no responses are mentioned corresponding to the proposed tests, i.e., on which quantities those tests will be used.**

A: We thank the reviewer for pointing this out. Due to an oversight on our part the Methods section of the abstract was garbled. This has been corrected. For the statistical tests that we plan to use, please refer to the Methods section.

**4. Longitudinal Design: From the current writeup, study doesn't look longitudinal (correct me if I am wrong!). Plans to consider a longitudinal study maybe crucial (can be part of the Discussion/Conclusion Section), given that those may reveal time-varying compositional changes between the two groups.**

**Reviewer #2: In the current manuscript, Nascimento et al. reported a registered study protocol aiming at evaluating the difference of microbiota in bile samples between healthy people and those with hepatobiliopancreatic diseases. The authors well described the study procedure in human samples and the processing of DNA preparation for sequencing. However, the authors did not well justify several factors including aging and gender which significantly contribute to the difference of microbiota profiles in their statistical analysis. Based on previous studies (Gut microbiota and aging, Science 04 Dec 2015:Vol. 350, Issue 6265, pp. 1214-1215; Intestinal Microbiota Is Influenced by Gender and Body Mass Index, PLoS One. 2016 May 26;11(5):e0154090.doi: 10.1371/journal.pone.0154090. eCollection 2016.), the proposed statistical analysis which did not consider age, body mass index, and gender as variance factors may not obtain significant outcomes.**

A: We appreciate your suggestion. Longitudinal studies would provide significant information regarding the variation of the microbiome composition of both groups over time, therefore, it is crucial to highlight the need to carry out this kind of study to better understand the microbiota of a site still so little known. This recommendation is best described in the Discussion.

Reviewer # 2's suggestion was also of great value to us to improve and enrich this study protocol. In Methods, in the field of statistical analysis, it is described that these possible confounding variables will be considered, which may, in fact, influence the composition of microbiomes, both in cases and controls.

Finally, we would like to thank you again for the points raised and we are at your disposal to clarify any doubts and make new changes, if necessary. We look forward to hearing from you regarding our submission.

Sincerely,

On behalf of all authors,

Alberto Meyer
